# Cost-effectiveness of introducing cone-beam computed tomography (CBCT) in the management of complex phalangeal fractures: economic simulation

**DOI:** 10.1007/s12306-020-00687-3

**Published:** 2020-11-19

**Authors:** N. Faccioli, E. Santi, G. Foti, G. Mansueto, M. Corain

**Affiliations:** 1 Department of Radiology, G.B. Rossi University Hospital, University of Verona, Piazzale L.A.Scuro 10, 37134 Verona, Italy; 2 IRCCS Sacro Cuore Don Calabria Hospital, Negrar, Verona, Italy; 3 Hand Surgery Department, G.B. Rossi University Hospital, Piazzale L.A.Scuro 10, 37134 Verona, Italy

**Keywords:** Cost-effectiveness, Cone-beam computed tomography, Complex phalangeal fractures, Finger, Multi-slice computed tomography

## Abstract

**Purpose:**

The purpose of this study was to evaluate the cost-effectiveness of introducing cone-beam computed tomography (CBCT) in the management of the complex finger fractures with articular involvement.

**Methods:**

We created a decision tree model simulating the diagnostic pathway of complex finger fractures, suggesting the use of CBCT as alternative to multi-slice computed tomography (MSCT), and we compared their clinical outcomes, costs, and cost-effectiveness for a hypothetical cohort of 10,000 patients. Measures of effectiveness are analysed by using quality-adjusted life years, incremental cost-effectiveness ratio, and net monetary benefit.

**Results:**

Diagnosis of a complex finger fracture performed with CBCT costed 67.33€ per patient, yielded 9.08 quality-adjusted life years, and gained an incremental cost-effectiveness ratio of 29.94€ and a net monetary benefit of 9.07 € at 30,000€ threshold. Using MSCT for diagnosis costed 106.23 €, yielded 8.18 quality-adjusted life years, and gained an incremental cost-effectiveness ratio of 371.15 € and a net monetary benefit of 8.09 €. CBCT strategy dominated the MSCT strategy. The acceptability curve shows that there is 98% probability of CBCT being the optimal strategy at 30,000€ threshold (1 EUR equal to 1.11 USD; updated on 02/02/2020).

**Conclusion:**

CBCT in complex finger fractures management is cost saving compared with MSCT and may be considered a valuable imaging tool in preoperative assessment, allowing early detection and appropriate treatment. It shortens the time to completion of diagnostic work-up, reduces the number of additional diagnostic procedures, improves quality of life, and may reduce costs in a societal perspective.

## Introduction

Finger fractures are frequent injuries, comprising 10–25% of all fracture-related visits in Emergency Department [1, 2]. The phalanges as a combined group are more commonly fractured (50%) than the metacarpals (42%). Proximal phalanx fractures are the most common (57.4%), followed by middle phalanx (30.4%) and metacarpal fractures (12.2%). They are the second most common fracture in young males with a mean peak age of 39.1 years [1–5]. Although most phalangeal fractures can be treated non-operatively with appropriate immobilization [3, 6, 7], complex finger fractures often need surgical management, especially those of proximal interphalangeal (PIP) joint, metacarpophalangeal (MCP) joint, and carpometacarpal (CMC) joint [5, 6]. The presence of displaced articular fragments, rotational misalignment, abnormal angulation and/or shortening, and soft tissue injury indeed may require a surgical approach [6–9]. Furthermore, an involvement of more than 25% of the articular surface represents an indication for surgery, in order to align the joint and minimize all that complications negatively influencing patients’ quality of life [7–13]. However, the final decision on the fracture’s management is based largely on clinical impairment of hand function, considering comorbidities, compliance, and the need to return to activities or work [6–8]. In this regard, conventional radiography (CR) represents the first-line imaging tool for the evaluation of metacarpal and finger fractures [14, 15]. Conversely, multi-slice computed tomography (MSCT) is not routinely performed in acute stage of injury [14, 15]. It can, however, be useful in assessment of articular surface involvement for detection of small bone fragments and in case of joint subluxation [6, 7, 10, 16]. Moreover, advanced imaging techniques are mandatory in case of equivocal findings depicted on initial radiographs [17–19]. In addition, malunion, delayed union, and non-union can be usually better assessed with MSCT [17–20]. Currently, MSCT is superior to CR in demonstrating intra-articular fracture lines and in the accuracy of fracture measurements (helpful in preoperative planning) because of slice thickness, isotropic, high-resolution multiplanar reconstruction (MPR) [16, 17, 21, 22]. However, benefits of CT devices are associated with the problem of radiation burden [23]. A possible approach for minimizing radiation doses is to implement cone-beam computed tomography (CBCT) technology [23, 24]. Furthermore, CBCT has been suggested as an alternative to MSCT for orthopaedic imaging to evaluate traumatic lesions of extremities [16, 18, 25, 26]. Advantages of CBCT include smaller set-ups, high spatial resolution, optimal reduction in metal artefacts, low radiation dose, and a relatively low cost of the equipment compared to MSCT [26]. Currently, there are very few reports of prospective studies comparing MSCT to CBCT for finger fracture characterization [18, 19]. Disadvantages of CBCT include higher radiation doses than conventional radiography, a limited contrast resolution, and the presence of various types of image artefacts [25].

The aim of this study is to investigate the cost and clinical effectiveness of CBCT as a diagnostic alternative in complex finger fractures compared with MSCT, using formal cost-effectiveness principles. We performed an economic simulation based on the assumption that the outcome could have a large economic impact on current practice due to the difference in cost of the imaging modalities, as well as the high incidence of finger fractures.

## Methods

### Target population

Target population includes a hypothetical cohort of 10,000 patients with complex finger fractures of an interphalangeal joint, visible on CR. We considered the baseline scenarios [27] of a 35-year-old patient with physical examination concerning for intra-articular fracture of an interphalangeal joint, visible on CR, and without surgical contraindications. Gender ratio was assumed to be 2:1 (male/female) because finger fractures have a higher incidence in young males [1–3]. Risk of misdiagnosis was included. For our base case, we assumed that the pretest probability for a finger fracture was 0.18 [1–8].

### Strategies compared

The strategies assessed in the model are: 1. CR then CBCT; 2. CR then MSCT. Using our base case, we first examined the optimal strategy. Then, we compared costs and QALYs among the two strategies.

### Study design and decision analysis model

**Fig. 1:**
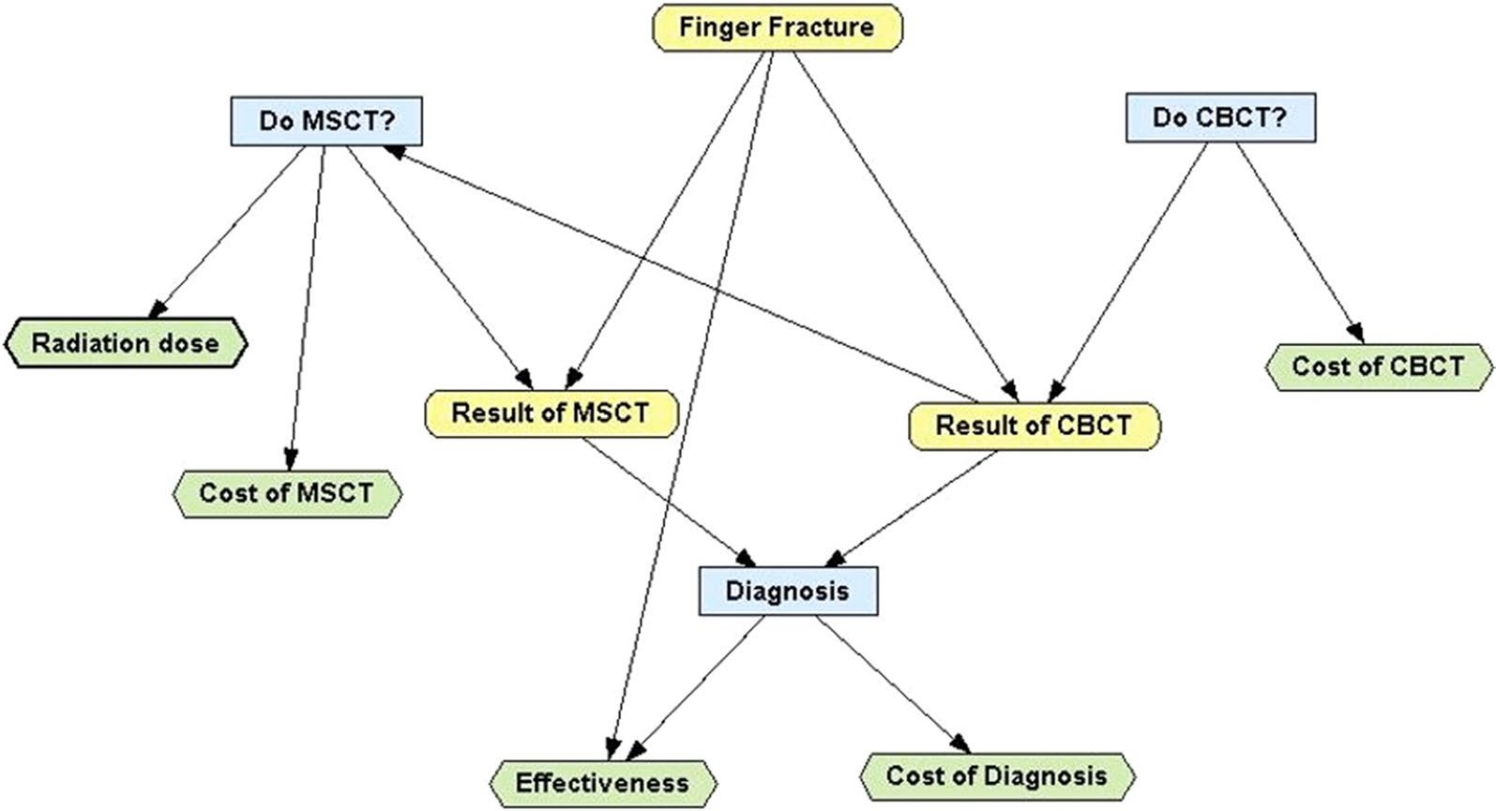
Decisional tree for management strategies in finger fracture

We conducted an economic-based simulation study using a decision analytic model, according to guidelines set by the Panel on Cost-Effectiveness in Health and Medicine [27]. This study is performed from the health care sector perspective, and we consider only direct costs of diagnostic tests. Cost-effectiveness analysis helps to evaluate whether the radiologic examination adds enough value to justify the costs. CR has limited capability to detect traumatic lesions, whereas CT is more sensitive. We tried to prospectively assess if CBCT imaging in addition to CR is cost-effective compared with the current diagnostic imaging strategy (CR, then MSCT) in patients with recent intra-articular injury of the fingers. We chose a decision tree as a one-period model in which branches represent chains of possible events, each with a certain probability of occurrence (Fig. 1). The entire cohort is distributed across the final health states (e.g. healthy, sick, and deceased), while each of these health states is associated with a certain amount of costs and health effects. This linear decision tree was created with the use of analysis software (OpenMarkov; CISIAD, UNED, Madrid, Spain) to compare two diagnostic strategies for a hypothetical cohort of 10,000 patients with complex finger fractures. In this model, all patients first underwent CR of the affected bone that comprised an anteroposterior (AP), lateral, and oblique view of the finger. The patients were randomized across two diagnostic strategies: CR followed, in the first week after trauma, by MSCT examination (1) or by CBCT examination (2). Exclusion criteria are age < 16 years or > 80 years and multiple fracture. The primary outcomes compared among the two strategies were QALYs (quality-adjusted life years), ICER (incremental cost-effectiveness ratio), and NMB (net monetary benefit). The widely used cost-effectiveness metric is the incremental cost-effectiveness ratio (ICER). This is calculated by dividing the difference in costs associated with two alternatives by the difference in QALYs.

The decision rule then applied is that if the ICER falls below a given cost per QALY threshold the result is cost-effective. If both costs are lower and QALYs are higher, the option is cost-effective. Net monetary benefit (NMB) is calculated by multiplying the total QALYs for a comparator by the threshold cost per QALY value and then subtracting the total costs. The comparator with the highest NMB is the most cost-effective option at the specified threshold. The highest NMB identifies the optimal strategy at a WTP of €30,000 per QALY gained (1 EUR equal to 1.11 USD; exchange rate updated on 02/02/2020). WTP of the Italian public health system for this type of follow-up could amount to €30,000/QALY, calculated on the average daily earnings in the country, based on Eurostat Statistics for 2017 [28, 29].

We set time horizon to 1 year. We conducted this economic evaluation within a modelling approach because it allows the synthesis of data obtained from meta-analyses, systematic reviews, and observational studies. The natural history of patients is showed by using a health state: cohort members were redistributed to different health states depending on the estimated probabilities of transition. Health effects were incorporated into the model by means of a long-term reduction in quality of life due to delayed treatment following a complication from a complex fracture. The model is a decision tree with four stages: fracture status; diagnostic strategy; diagnostic result; and cost-effectiveness of diagnosis.

### Quality of life

To calculate the total QALYs for each diagnostic strategy, we incorporated a range of health-related quality-of-life estimates based on previously published data regarding hand function. The most used tool evaluating the hand function described in the literature is DASH score (Disability of Shoulder, Arm and Hand Questionnaire) [30].

DASH questionnaire evaluates physical impairment with five answer options for each item. These utility instruments in the form of a questionnaire represent a single health state by documenting several domains, representing none, some, or extreme problems in that area. Health gains were showed as quality-adjusted life years (QALYs). If the fracture, identified on imaging, is treated successfully and a good functional outcome is achieved without any complications, the patient will return to the general population utility. If complications occur (stiffness, non-union, infection, and nerve injury) and the functional outcome is low, they will remain in the initial reduced health state for the remaining years of life [31]. The utilities are summed across the lifetime for all patients along each diagnostic pathway. To derive a utility from the DASH score, we predicted the EQ-5D index for the DASH score using a mapping function. In the literature, we did not find mapping studies for these outcomes, so we developed our own using data from a population with distal radial fractures [32]. The variables used to predict the EQ5D score were the pre-injury EQ5D score, DASH pain component score, the function component of the EQ5D, and the product of the components. The model applies the utility score calculated from the mapping, to each person with a finger fracture. QALYs are calculated by multiplying the duration of time spent in a health state by this utility score associated with that health state.

### Sources of probabilities and cost estimates

Table 1 lists all parameters of the model. Direct medical costs, analysed from a societal perspective, included costs of diagnostic procedures, calculated considering the initial investment of equipment, additional costs during use, maintenance costs, years of use, personnel costs, and materials used (provided by the Hospital Technical Department) [33]. Total costs were 32.00 € for CR; 67.33 € for CBCT; and 106.23 € for MSCT. The prevalence of finger fracture and performance characteristics of imaging studies were derived from published information [1, 2, 18, 19]. Performance characteristics and utility values of cross-sectional imaging were derived from published information: MSCT sensitivity in the diagnosis of finger fractures is 94% and specificity is 92% [18–21]. Sensitivity and specificity for CBCT amount to 91% and 93%, respectively [18, 19, 22, 25–27]. The mean age of the population of people with complex finger fractures was assumed to be 35 years. This was built on the mean ages assumed by the included clinical studies, which ranged from 22 to 39 years. Life expectancy was estimated from Eurostat Statistics Life Tables and was estimated for a 30-year-old as 49.87 remaining years [28]. This gives a mean age at death of 80 years.

**Table 1:** Parameters used in Markov model

Parameters	Baseline estimate	Reference no.
Prevalence of finger fracture	10–25%	1, 2, 4
Pretest probability of a fracture	1.5–2%	1–6
*Baseline risk*		
Correct diagnosis	0.98	18,19
Probability that the fracture is displaced	0.12	10–13, 42,43
Increased prevalence of non-union for intra-articular fracture	0.15	10–13, 42,43
Increased prevalence of arthritis for intra-articular fracture	0.784	10–13, 42,43
*Tests performance characteristics*		
Sensitivity of conventional radiography	0.70	15, 17, 20
Sensitivity of MSCT	0.90	16, 18–22
Specificity of MSCT	0.98	16, 18–22
Sensitivity of CBCT	0.96	17–19, 22, 26–28
Specificity of CBCT	0.90	17–19, 22, 26–28
*Unit costs*		
Cost of plain film X-ray	32 €	38
MSCT	106.23 €	38
CBCT	67.33 €	38
*Health utility*		
Base case	0.85	
General population QoL for 30-year-olds	0.93	1,2,4,30
Duration of reduced QoL for complication		
From complex fractures	Lifetime	10, 32–37
Mean age at time of injury	35 years	10, 32–37
Mean age at death	80 years	10, 32–37
Immobilization	0.759	32–34
Symptoms (instant decrease)	−0.03	32–34

CBCT: cone-beam computed tomography. MSCT: multi-slice computed tomography. QoL: quality of life

### Data analysis

Using our base case, we first observed the optimal strategy with both MSCT and CBCT capabilities and we compared costs and QALYs among the two strategies. We based our probabilistic sensitivity analysis on the second-order distributions assigned to some parameters, carried out by means of stochastic simulations (Monte Carlo techniques) [27, 34, 35]. To evaluate the decision, we perform deterministic one-way sensitivity analysis (tornado diagrams and spider diagrams) to determine how the pretest probability of a fracture affected our outcome and to calculate the threshold for which the optimal strategy remains cost-effective. Then, we perform a multiway probabilistic sensitivity analysis on all input parameters simultaneously. The way in which distributions are defined reflects the nature of the data: for example, utilities are given a beta distribution, which is bounded by 0 and 1, reflecting that a quality-of-life increment will not be out of this range. Costs are given in a gamma distribution. The following variables were left deterministic (they were not varied in the probabilistic analysis): cost-effectiveness threshold, sensitivity of MSCT, specificity of MSCT, sensitivity of CBCT, specificity of CBCT, general population quality of life, duration of reduced quality of life, mean age at time of injury, and mean age at death. All the variables that were made probabilistic in the model and their distributional parameters are detailed in Table 1. Many deterministic sensitivity analyses are engaged to evaluate the robustness of model assumptions. In these analyses, one or more inputs were changed, and the analysis rerun to evaluate the impact on results and whether conclusions on which intervention should be recommended would change.

## Results

### Baseline analysis

Given the base case assumptions, diagnosis of a complex finger fracture performed with CBCT costed 67.33€ per patient, yielded 9.08 QALY, and gained an ICER of 29.94 €/QALY and an NMB of 9.07 €. Using MSCT for diagnosis costed 106.23 €, yielded 8.18 QALY, and gained an ICER of 371.15 €/QALY and an NMB of 8.09 € at 30,000€ per QALY threshold (Table 2). Incremental NMB is 1.17 for CBCT strategy and 0.19 for MSCT strategy. For the base case, CBCT is identified as optimal strategy due to the highest NMB at a WTP of 30,000 € per QALY gained. The MSCT strategy was dominated. Using the ICER decision rule, we can see that the most cost-effective option is CBCT, and all other options are dominated. The plot of cost versus effectiveness we obtained shows that MSCT is dominated by CBCT because the latter is cheaper and more effective.

**Table 2:** Results of probabilistic sensitivity analysis for each management strategy

	Conventional radiography	CBCT	MSCT
Cost per patient (€)	32	67.33	106.23
QALYs per patient	7.9	9.08	8.18
ICER	Reference	29.94	371.15
NMB at 30,000€ per QALY threshold	7.89	9.07	8.09
Incremental NMB	/	1.17	0.19
Ranking	3rd	1st	2nd

QALY: quality-adjusted life years, ICER: incremental cost-effectiveness ratio, NMB: net monetary benefit

### Deterministic unicriterion sensitivity analysis

One-way sensitivity analysis showed that CBCT is the optimum strategy. Tornado diagrams are useful as deterministic sensitivity analysis tools comparing the relative importance of variables. The tornado diagram we obtained shows that “Prevalence of finger fracture” and “Cost of CBCT” are the parameters having the highest impact on the expected utility. They are the most influential assumptions; consequently, a decrease in the cost of the tests makes the expected utility increase.

**Fig. 2:**
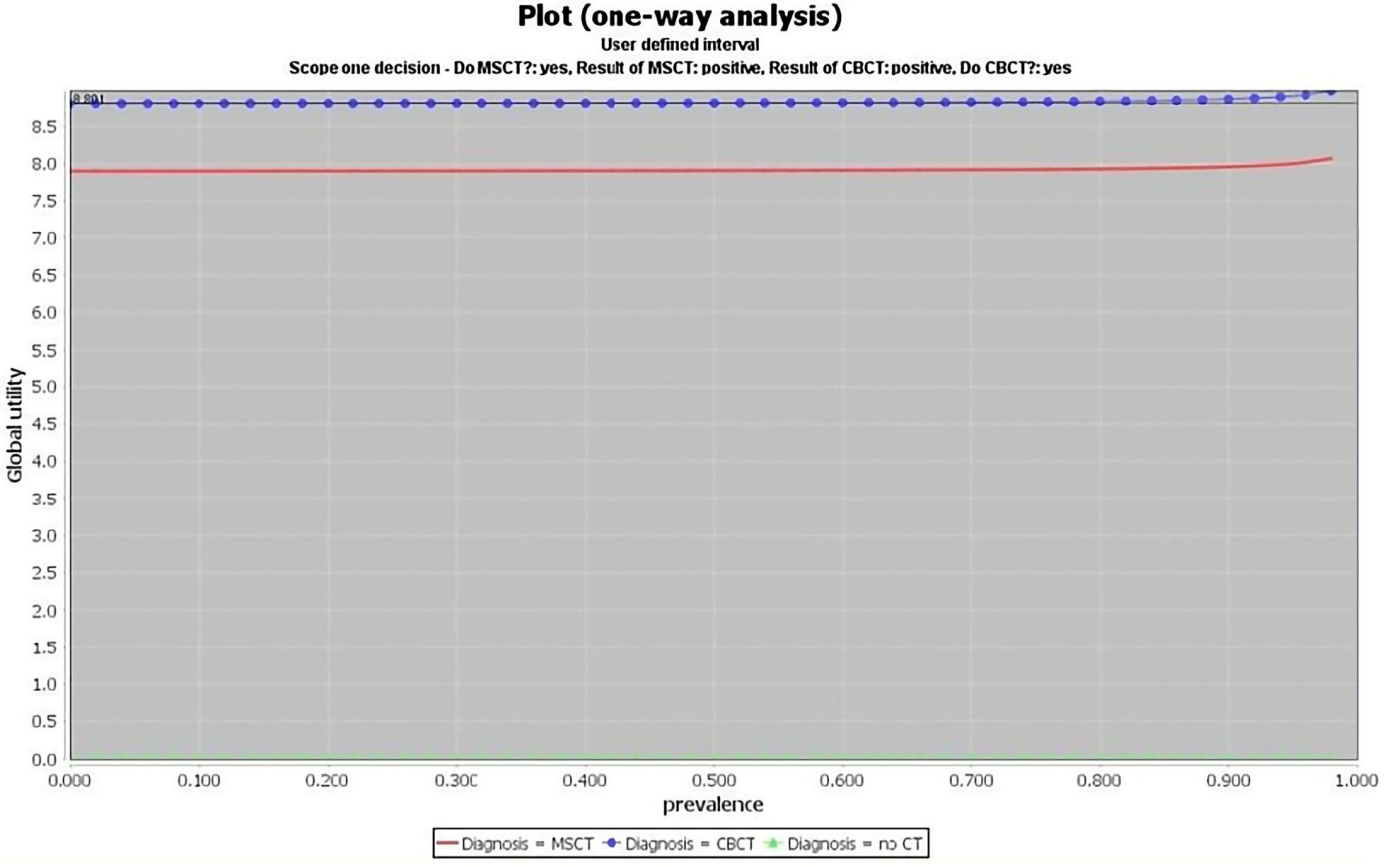
One-way analysis plot for the decision “Diagnosis”. The black horizontal line denotes the expected utility in the reference case. The red line denotes the effectiveness of MSCT, the blue line the effectiveness of CBCT, and the green line the effectiveness without CT. Effectiveness results 8.18 for MSCT and 9.08 for CBCT; the latter is the best choice

The assessment of how the expected utility can change among the parameters is another type of deterministic unicriterion analysis. Analysing Fig. 2, we consider a scenario for which there is no evidence; therefore, the probability of the disease and its prevalence correspond. The interpretation of this plot is as follows. The black horizontal line denotes the expected utility in the reference case, 8.891. When the prevalence of the disease is 0, we are sure that the disease is absent. In this case, the effectiveness of MSCT (red line) is 7.9 and the effectiveness of CBCT (blue line) is 8.8. When the prevalence is 1, we are sure that the patient has the disease. Then, the effectiveness is 8.18 for MSCT and 9.08 for CBCT; the latter is the best choice. The plot indicates an effectiveness of 8.18 for MSCT and 9.08 for CBCT. The last is the most suitable choice, according to Table 1.

### Probabilistic cost-effectiveness sensitivity analysis

Starting from the incremental cost-effectiveness ratio, we built an acceptability curve, which is a graphical representation of the results. The curve shows the percentage of points with a lower cost-effectiveness than the WTP itself, that is the cost threshold. If WTP is less than €7,500/QALY, the best option is not to perform CT. If WTP is €10,000/QALY, CBCT is the optimal strategy in 90% of cases, while MSCT is the optimal strategy in 5%. All these data are derived from various sources with different reliability and are affected by different levels of uncertainty, expressed as standard error or confidence interval. Given that this uncertainty can be moderated, we created acceptability curves generated through the probabilistic sensitivity analysis. We assigned a probabilistic theoretical distribution for every parameter (beta or gamma), and we let them propagate through a Monte Carlo simulation. The analysis was conducted by running Markov model 10.000 times, and each time the probability of transition was different because it was extracted from the distribution.

## Discussions

This economic analysis evaluated the most clinically and cost-effective imaging strategy for patients presenting with a complex finger fracture. As imaging modalities improve, reassessing diagnostic algorithms, considering imaging performance and testing cost, is mandatory to achieve cost-effectiveness. Historically, this decision-making process has been dominated by concern for the negative sequelae of a non-union fracture [36, 37] and, when initial radiographs are equivocal or in case of complex fractures (articular involvement or fragments angulation–dislocation), further multiplanar, high-resolution imaging with CT can be needed [16, 17, 20, 21]. The choice between CBCT and MSCT may be justified by costs and performance characteristics: CBCT is a more cost-effective diagnostic modality due to high sensitivity and lower costs compared with MSCT [17–19, 22]. The sensitivity and specificity of MSCT and CBCT are both very high. Based on the costs and performance characteristics used in this study, CBCT may be a more cost-effective diagnostic modality due to high sensitivity at decreased costs compared with MSCT. The cost-effective plane shows that both CBCT and MSCT are clinically beneficial; however, MSCT lies in the area above the horizontal in the upper right-hand quadrant (it is cost-increasing), whereas CBCT lies in a lower position (more effective and cost-saving). In this cost–effectiveness analysis, obtaining a CBCT was more convenient than performing MSCT when assessing a complex finger fracture, with a 36.61% saving.

In this model, CBCT imaging represents the dominant strategy in all scenarios. Furthermore, CBCT appears to be more cost-effective at a much lower willingness-to-pay (WTP) threshold than MSCT. The decision-maker’s WTP threshold for each finger fracture detected can affect the choice of the optimal strategy. For example, if we accept the threshold of € 10,000 per finger fracture detected, CBCT is the optimal strategy, whereas not to perform CT is more appropriate at lower levels of WTP. For a WTP of €30,000/QALY, there is 98% probability of CBCT being the optimal strategy and there is 2% probability of MSCT being optimal strategy. According to our results, when evaluating finger fractures, CBCT should be preferred to MSCT both from a clinical and from a economical perspective. CBCT is a readily available, fast examination that helps in shortening the time to completion of diagnostic work-up. Allowing a precise and reliable diagnosis, CBCT reduces the number of additional diagnostic procedures, improving the quality of life and reducing the costs in a societal perspective. Additionally, a recent review of CBCT musculoskeletal clinical applications [25, 38] highlighted the limited cost of purchase, installation, and maintenance and recommends its use in centres with a high turnover of musculoskeletal procedures or as an additional imaging tool in large hospitals. Another hypothetic advantage of CBCT over MSCT is that the first one, being usually employed for outpatient examinations, should be easily available. Conversely, MSCT, employed for acute traumatic and non-traumatic patients, including major traumas, may be not readily available for the evaluation of finger fractures. Moreover, CBCT images provide essentially equivalent diagnostic information to MSCT images at a lower radiation dose [24, 25]. There is no statistically significant difference between CBCT and MSCT with respect to diagnostic accuracy for finger fractures, although the radiation exposure for CBCT is 1/3 that of MSCT [18, 19, 37]. Kröpil et al. compared CBCT measurements of bony bridging with histologic findings in mini-pig models and found that CBCT can accurately quantify bone healing [39]. CBCT can minimize radiation doses in 3-D imaging of extremities, when compared with MSCT modalities. In a recent study, Koivisto et al. showed 21.4 μSv effective dose for a standard MSCT protocol, between a 1.9 and 4 μSv effective dose for standard CBCT protocols and 1.5 μSv using CR [24]. Moreover, CBCT may be helpful in situations where occult fracture or non-union is suspected, the fracture is in a region with multiple overlapping structures, or an overlying splint or cast cannot be removed.

Although CBCT is widely used in medical field, it has some inherent limitations. Image quality can be reduced due to artefacts, noise, and poor soft tissue contrast. Artefacts limit an accurate visualization of soft tissues; they can be due to beam hardening, to patient motion, or be related to the scanner or cone beam (partial volume averaging, under-sampling). The wider collimation of CBCT results in an increase in scattered radiation, contributing to image degradation and to decrease in the signal-to-noise ratio [25].

Our study has several limitations. This analysis is purely theoretical and relies on probability values taken from heterogeneous sources of literature. Moreover, the model requires several assumptions: accordingly, results depend on these data inputs. Based on the available literature, for example, we assumed that the possible pretest probability range for a finger fracture was 1.5–2%, for the one-way sensitivity analysis [1–6]. This probability depends on clinical judgement and experience of the first-aid physician and on the patient’s symptoms [4–8]. However, to test these assumptions, we conducted sensitivity and probabilistic to establish the robustness of our results. Additionally, we assumed that clinical outcomes were the same for all identified fractures treated and that patients would return to full health after 1 year. The inclusion of these patients would raise the cost and morbidity of the empiric treatment group, which would amplify the advantage of immediate advanced imaging. Like any model, the algorithm used in our study cannot encompass every clinical scenario that might be faced by the clinician. It is important to place this study in the Italian health care context: as a result, cost will be unique. Our aim was to provide information on possible compromises for this problem and to clear the way to new possible guidelines. Each system would need to employ local cost data to determine individual savings.

## Conclusions

In conclusion, we found that for hospitals with access to both CBCT and MSCT scanners, the use of CBCT for the radiological assessment of complex finger fracture represents the most cost-effective strategy. In the hypothesis of complex finger fracture indeed, CBCT is more cost-effective than MSCT imaging and is nearly as cost-effective as traditional conventional radiology diagnosis.

## Data Availability

Not applicable.

## Abbreviations

CT: Computed tomography
CBCT: Cone-beam computed tomography
MSCT: Multi-slice computed tomography
QALY: Quality-adjusted life year
ICER: Incremental cost-effectiveness ratio
NMB: Net monetary benefit
iNMB: Incremental net monetary benefit
QoL: Quality of life
